# Correction Notice: Establishment of Human PD-1/PD-L1 Blockade Assay Based on Surface Plasmon Resonance (SPR) Biosensor 

**DOI:** 10.21769/BioProtoc.4844

**Published:** 2023-08-20

**Authors:** Tess Puopolo, Huifang Li, Justin Gutkowski, Ang Cai, Navindra P. Seeram, Hang Ma, Chang Liu

**Affiliations:** Department of Biomedical and Pharmaceutical Sciences, College of Pharmacy, University of Rhode Island, Kingston, RI 02881, USA;

In the Background section of “Establishment of Human PD-1/PD-L1 Blockade Assay Based on Surface Plasmon Resonance (SPR) Biosensor" (https://bio-protocol.org/e4765), “Alsaab et al., 2018” is incorrectly referenced. The correct citation is “Alsaab et al., 2017”. The sentence should read as follows: “Clinical data have demonstrated that the blockade of PD-1 or PD-L1 can boost T cell–mediated antitumor responses, generates durable clinical responses, and prolongs patient survival time (Ohaegbulam et al., 2015; Alsaab et al., 2017).”

